# Primary Hyperparathyroidism: Is Image Localization Alone Sufficient to Ensure Long-Term Cure in Unifocal Disease?

**DOI:** 10.7759/cureus.31244

**Published:** 2022-11-08

**Authors:** Joseph Mathew, Ravi Arjunan, Syed Althaf, Rajshekar Halkud

**Affiliations:** 1 Department of Surgical Oncology, Kidwai Memorial Institute of Oncology, Bangalore, IND; 2 Department of Head and Neck Oncology, Kidwai Memorial Institute of Oncology, Bangalore, IND

**Keywords:** primary hyperparathyroidism, parathyroid adenoma, parathyroidectomy, ultrasound, sestamibi

## Abstract

Introduction: Advances in imaging have facilitated precise preoperative localization and focused resection of hyperfunctional parathyroids in primary hyperparathyroidism (PHPT). Combining imaging techniques or a “dual” approach, when concordant, improves adenoma-localizing accuracy above individual modalities. This study sought to assess biochemical cure and failure rates (persistence or recurrence) of surgery directed by dual imaging alone in PHPT.

Methodology: This observational, single-center analysis comprised 31 patients diagnosed with PHPT and imaged with both ultrasound (USG) of the neck and sestamibi scintigraphy. The extent of surgery was based solely on inter-modality concurrence for adenoma localization; imaging-concordant patients underwent focused parathyroidectomy, whereas discordant patients necessitated neck exploration (with extent altered according to scintigraphic lesion lateralization). No intraoperative localizing adjuncts were used.

Results: Twenty-three patients had concordant imaging, of which 19 underwent focused exploration, with sensitivity and positive predictive value (PPV) for dual imaging of 100% and 95.7%, respectively. The overall sensitivity and PPV were 92.9% and 89.7% for USG alone and 100% and 93.6% for scintigraphy, respectively. The mean age and prevalence of thyroid disease were significantly higher in the discordant group. All patients achieved postoperative normocalcemia. There were no cases of persistent or recurrent hyperparathyroidism on follow-up.

Conclusions: In the imaging-concordant setting, focused surgery may be safely performed with the omission of other adjuncts for localization. Older age and concomitant thyroid pathology predispose to discordant imaging and are risk factors for surgical failure when attempting an image-directed approach. Neck exploration is an alternative in these patients with excellent cure rates and acceptable morbidity.

## Introduction

Surgery for primary hyperparathyroidism (PHPT) has evolved from the traditional bilateral neck exploration (BNE) to the current practice of minimally invasive parathyroidectomy (MIP). A better understanding of disease etiopathogenesis, particularly its unifocality, in the vast majority, allowing for a focused exploration, along with the development of more precise imaging modalities and intraoperative adjuncts to localize abnormal glands, has driven this change such that cure rates with MIP approach 99% in high-volume centers [1]. Accurate preoperative localization is vital for the success of MIP, which involves the excision of abnormal parathyroid(s) via a 2-2.5 cm cervical incision immediately over the dysfunctional gland(s) [2].

Most centers today perform combination imaging for localization with dual-modality ultrasound (USG) and sestamibi scintigraphy being the most widely used. When concordant, accuracy rates for locating unifocal disease approach 95% [3]. However, these figures decrease significantly in discordance and in multiglandular dysfunction, predisposing to persistent disease when image-directed MIP is attempted [3,4].

Adjuncts such as intraoperative parathyroid hormone (IOPTH) assays were introduced to reduce failure rates. Success rates of 87%-99% were reported with IOPTH-directed surgery in unifocal disease, although, as with imaging, sensitivity dropped significantly with multifocality (58%) [3,5]. Despite being incorporated into PHPT management protocols worldwide, IOPTH facilities are not universally available and add significantly to operating time and costs [3,6].

This study was conducted to assess the feasibility and outcomes of surgery directed by dual imaging alone without the use of intraoperative adjuncts in the management of PHPT in terms of biochemical cure and disease persistence or recurrence.

## Materials and methods

This observational, single-institution study on sporadic PHPT was conducted between September 2015 and August 2021 in the Department of Surgical Oncology and Head and Neck Oncology services at Kidwai Memorial Institute of Oncology, a tertiary cancer center in South India. Consecutive patients with a biochemical diagnosis of PHPT (raised PTH in the setting of hypercalcemia) regardless of etiopathogenesis (adenoma, hyperplasia, or carcinoma) or disease focality, without prior history of neck surgery or history suggesting familial or hereditary disease, who had been evaluated with both USG and sestamibi scintigraphy and had undergone image-directed parathyroidectomy at the Institute were included. Informed consent was obtained from all patients prior to surgery.

As per Institute protocol, serum calcium, albumin, and PTH of all patients with suspected PHPT were initially evaluated, followed by scintigraphy with USG of the neck after diagnosis. Sestamibi scintigraphy or technetium-99m-sestamibi (99m Tc-MIBI) parathyroid scan involved the injection of 20 mCi Tc-MIBI, followed by the acquisition of static anterior cervical and thoracic images at 10, 60, and 120 minutes with a gamma camera. Foci of increased tracer uptake, persisting on late sequences, were considered a positive scan. USG of the neck was performed using a 12-MHz high-frequency probe with parathyroid disease characterized by hypoechogenicity, increased vascularity, mobility, and relation to the thyroid. Concomitant thyroid pathology, when present, was noted. Findings were verified by two radiologists, at least one of them being a senior consultant.

With regard to the localization of abnormal glands, an agreement between the two modalities was considered imaging “concordance,” and these patients underwent focused parathyroidectomy with no attempt made to explore the other glands. In the imaging-discordant group, patients who had had their lesions lateralized on scintigraphy underwent unilateral neck exploration (UNE). Negative imaging or failure of intraoperative lesion localization (with the above approaches) necessitated conversion to BNE. The extent of the incision, when required, was also modified to address any associated thyroid disease.

Intraoperative findings, including location and (if documented) size of the adenoma, and status of the other glands (if explored) were noted. In case of uncertainty, a frozen section analysis was sought to differentiate parathyroid from non-parathyroid tissue. No intraoperative localizing adjuncts were used. The results of preoperative localization studies were correlated with the intraoperative findings (in terms of the location of the lesion and the status of other glands when visualized) and the disease pathology to assess the accuracy of these imaging modalities both individually and combined. Concordance of imaging with these parameters was considered a “true positive” result.

Complications encountered on-table and in the immediate postoperative period were recorded. Serum calcium was obtained 24-36 hours after surgery, with the endpoint for surgical success being euglycemia (normal range: 8.5-11.5 mg/dL), and was repeated only for clinically, or biochemically, evident calcium imbalance. Serum PTH was not repeated postoperatively. All patients were followed up with serial serum calcium levels for at least six months with sustained euglycemia during this period defining a biochemical cure. Telephone interviews were also conducted to assess for any symptoms suggestive of disease persistence or recurrence.

Statistical analysis was performed using R software version 4.0.2. Continuous variables (presented as mean and standard deviation) were compared using an independent Student’s t-test (for two groups). Pearson’s correlation was applied to determine the direction and strength of linear relationships between continuous variables. Categorical variables were expressed as counts and percentages. The chi-square test was used to identify associations between these. When the expected cell count was less than 5, Fisher’s exact test was performed. A p-value of <0.05 was considered statistically significant.

## Results

During the study period, 41 patients with PHPT were identified, of which 10 were excluded: three on the account of previous neck surgery, three for deviations in the protocol, and four with incomplete records and/or documentation. The remaining 31 patients comprised the study cohort (Table 1).

**Table 1:** Comparison of demographic and biochemical parameters, diagnostic accuracy of imaging, surgical management, and postoperative outcomes in the overall patient population and in the two study groups. PTH: parathormone; PPV: positive predictive value; USG: ultrasound of the neck

			Overall (n=31)		Comparison of the study groups				p-value
					Concordant (n=23)	Discordant (n=8)			
Mean age in years			39.2±14.99		34.7±11.66	52.1±16.67			0.003
Gender	Male		6 (19.4%)		5	1			1.000
	Female		25 (80.6%)		18	7			
Mean serum calcium in mg/dL			13.8±1.86		13.6±1.72	14.3±2.25			0.372
Median PTH in pg/mL (range)			557.6 (77.5-2,205.1)		542 (77.5-2,205.1)	636.9 (95.3-1,229)			0.630
Presence of thyroid disease			9 (29%)		4 (17.4%)	5 (62.5%)			0.027
			Overall (n=31)		Concordant (n=23)	Discordant (n=8)			p-value
Imaging			Sestamibi	USG	Sestamibi+USG	Sestamibi		USG	
True positive			29	26	22	Side localized	3	4	
						Site localized	4		
False positive			2	3	1	1		2	
False negative			0	2	0	0		2	
Sensitivity			100%	92.9%	100%	100%		66.7%	
PPV			93.6%	89.7%	95.7%	87.5%		66.7%	
Surgery		Focused	18		18	-			
		Unilateral	8		2	6			
		Bilateral	5		3	2			
Transient hypocalcemia			10		6	4			0.381
Median follow-up in months (range)			21 (1-75)		21 (2-63)	25.5 (1-75)			0.784

The most common age group affected was the fourth decade (32.3%), with 61.3% under 40 at presentation. Most patients presented with constitutional and musculoskeletal symptoms (58.1%). A significant negative linear relationship was noted between the duration of symptoms and serum calcium at presentation (correlation coefficient=-0.475; p=0.007).

All patients had positive scintigraphy, of which 23 (74.2%) were concordant with USG and hence candidates for focused parathyroidectomy. However, four had concomitant thyroid disease requiring surgery and hence an extension of incision for access. Subsequently, 19 patients underwent a focused exploration with the adenoma successfully identified and excised in 18 (sensitivity; 100%; PPV: 95.7%). In one patient, a negative focused exploration necessitated a BNE (false-positive localization; conversion rate: 5.3%). In the discordant-imaging group (25.8%), based on side localization on scintigraphy, UNE was planned. However, four patients required surgery for thyroid disease (including two for total thyroidectomy), and exposure was tailored accordingly. In this subgroup, one patient was noted to have a false-positive scintigraphy finding, with an adenoma detected on the opposite side. Frozen section analysis was sought in 51.6% of patients at the discretion of the operating surgeon.

Overall, USG alone was able to identify the gland in 83.9%, with an overall sensitivity and PPV of 92.9% and 89.7%, respectively. However, in cases of discordance, these values dropped to 66.7%. Scintigraphy, on the other hand, had an overall sensitivity of 100% and PPV of 93.6%; a marginal decrease in PPV to 87.5% was noted in the discordant group.

Biochemical cure was achieved in all 24 patients. However, 10 developed transient hypocalcemia (biochemical or clinical), not associated with the extent of surgery (p=0.766). There were no cases of permanent hypocalcemia. One patient with concomitant PTC developed unilateral recurrent laryngeal nerve (RLN) palsy post-total thyroidectomy. The median duration of follow-up was 21 (range: 1-75) months. Among those on surveillance, there have been no cases of persistent or recurrent disease to date.

## Discussion

Although BNE has long remained the gold standard procedure for PHPT, MIP has emerged as the more popular alternative, with significant reductions in operating time, rates of postoperative pain, and severe complications (including symptomatic hypocalcemia), translating into a shorter hospital stay, reduced cost of treatment, and better cosmesis, without compromising cure rates [1,2,6]. However, accurate preoperative localization of involved glands is vital for success. Studies utilizing USG and scintigraphy for localization have reported accuracy rates of 82.8% for sestamibi scintigraphy in lateralizing the lesion and 79.5% for USG rising to 87.5% when both modalities were concordant [7]. In this series, USG had an overall sensitivity of 92.9% and 100% when concordant. PPV was 89.7% and 95.7%, respectively, possibly due to experienced operators, availability of cues (scintigraphy performed prior to USG), and larger size of adenomata excised (although this data was not uniformly available in our cohort).

**Figure 1:**
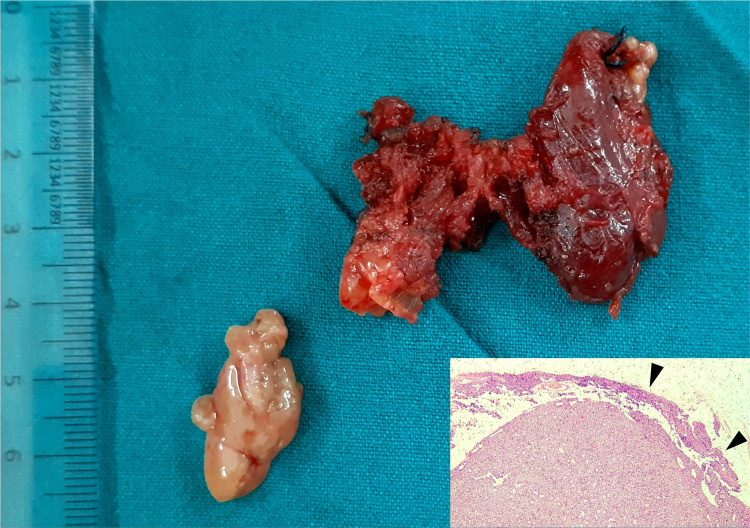
Concomitant parathyroid adenoma with papillary thyroid carcinoma in a 42-year-old female, necessitating total thyroidectomy with right inferior parathyroidectomy. The size of the lesion and its adherence to the thyroid led us to suspect parathyroid carcinoma; however, the histopathology (inset) was suggestive of an atypical adenoma with an intact capsule (black arrowheads).

Thyroid nodules compromise imaging accuracy by obscuring or mimicking parathyroid pathology and may also preclude a focused approach even when feasible. In a study evaluating the feasibility of MIP in a region endemic for thyroid disease, 49% of patients with solitary adenoma-related PHPT were found to have associated goiter. Concomitant thyroid disease was found to reduce the sensitivity of sestamibi and USG from 89% and 86% to 83% and 70%, respectively [4]. Noted in nearly 30% of patients on imaging in our study, thyroid disease was significantly more common in the discordant group, with an associated reduction in ultrasound accuracy. Six patients merited operative intervention: two on the account of indeterminate nodules, two for multinodular goiter, and, interestingly, two for papillary thyroid carcinoma (Figure 1).

Thus, concomitant thyroid pathology requiring surgery could be considered a potential confounding factor both in terms of affecting imaging accuracy and when assessing the adequacy of focused parathyroidectomy for adenoma excision, as ipsilateral parathyroids are identified during thyroid surgery. The mean patient age in the discordant group was also significantly higher, an indication of the increasing prevalence of incidental thyroid nodules in older populations.

Hence, older age and concomitant thyroid disease may be considered risk factors for discordance on imaging predisposing to surgical failure, and these may be potential candidates for intraoperative adjuncts. In India, patients with PHPT are typically younger (with an associated lower prevalence of thyroid pathology) and present late with florid manifestations and striking biochemical abnormalities. The accuracy of imaging in this setting would be superior. The sensitivity of sestamibi is known to increase with increasing serum calcium and PTH levels; over 90% of patients with positive scans harbored levels greater than 11.3 mg/dL and 160 pg/mL, respectively [8].

IOPTH was introduced as a means to document the completeness of surgery on-table. However, it has been shown to be less beneficial when imaging is concordant. In a large study, a significant rate of both false negatives (5.5%) and positives (2%) were noted with PTH assays. The failure rate of MIP was 2.6%, which was entirely due to the erroneous localization of dysfunctional glands. The authors concluded that IOPTH would increase cure rates by only 1%-2% when used in conjunction with other localization techniques [9].

Hence, the main indications for PTH assays intraoperatively would be in the setting of multiglandular disease, familial syndromes, discordant or negative functional imaging, and possibly, the recurrent setting, wherein IOPTH may increase surgical success rates to 94% [6]. It is important to remember that IOPTH only predicts residual disease without localization, and a persistently raised IOPTH should be considered an indication for further dissection. This extended surgery, which is essentially a blind procedure liable to result in RLN injury or parathyroid devascularization, requires the surgeon to explore and identify the other glands [9]. However, would morphology match functionality? How reliable would clinical judgment be in deciphering normal from abnormal glands based on appearance? These points and the possibility of overlooking ectopic glands, however rare, should be kept in mind when IOPTH does not drop significantly after adenoma excision, bringing the onus back on comprehensive imaging to detect disease [10,11].

Although this study was observational, the homogeneity of the patient cohort was controlled to the extent that it was single-center with strict inclusion criteria, uniform protocol, and a short study period. Moreover, PHPT in India is rare, with only overtly symptomatic patients seeking medical attention in the absence of screening, reflecting the small sample size. A significant proportion of patients had concomitant thyroid pathology, which may have confounded our results with regard to imaging accuracy and the adequacy of MIP in achieving a cure for PHPT. Despite these limitations, the study reflects the constraints faced by surgeons in the developing world and opens up new avenues of research to maximize the usage of resources in a resource-constrained setting.

## Conclusions

Concordance on dual imaging correlates strongly with intraoperative localization of parathyroid adenomata, and in this setting, other localizing adjuncts may potentially be foregone. In imaging-discordant cases, in the absence of intraoperative tools for localization, UNE may be a viable alternative to BNE with acceptable morbidity when the laterality of the hyperfunctioning gland is known by scintigraphy. Older age and concomitant thyroid pathology are risk factors for discordance in imaging and should alert the surgeon of a higher likelihood of surgical failure with image localization alone. Larger multicentric studies evaluating long-term cure rates and the cost-effectiveness of image-directed MIP will help identify the subset of patients most likely to experience successful outcomes with this approach in resource-limited settings.
